# Essentials of Medical and Clinical Chemistry

**Published:** 1900-11

**Authors:** 


Essentials of Medical and Clinical Chemistry. With Laboratory Exercises. By Samuel E. Woody, A. M., M. D. Fourth edition, revised and enlarged. Illustrated. Philadelphia: P. Blakiston's Son & Co. 1900. [Price, $1.50.]

This little volume is a model of excellence. Nothing essential to a proper knowledge of medical chemistry is omitted, yet on the other hand the student is spared the burdensome details of that science which, in the words of Dr. Holmes, is so liable to spoil on one's hands. The fourth edition has been revised and largely rewritten.

